# Generational Patterns in Work Engagement, Work Ability, Job Satisfaction and Turnover Intentions Among Nurses: A Cross‐Sectional Study

**DOI:** 10.1155/jonm/9713267

**Published:** 2026-08-19

**Authors:** Dania Comparcini, Eleonora Paro, Giuseppe Tonutti, Michele Chittaro, Mariagrazia Valentini, Rosanna Finos, Marco Tomietto

**Affiliations:** ^1^ Section of Nursing, Interdisciplinary Department of Medicine, “Aldo Moro”University of Bari, Bari, Italy; ^2^ Azienda per L’Assistenza Sanitaria “Friuli Occidentale”, Pordenone, Italy; ^3^ School of Healthcare and Nursing Sciences, Centre for Health and Social Equity (CHASE), Northumbria University, Newcastle-upon-Tyne, UK, northumbria.ac.uk

**Keywords:** engagement, job satisfaction, nursing, turnover intentions, work ability

## Abstract

**Aim:**

To examine generational patterns in work engagement, work ability, job satisfaction and internal and external turnover intentions among nurses in a hospital and community healthcare setting, and to examine whether those patterns persist after controlling for sociodemographic and occupational variables.

**Design:**

Cross‐sectional.

**Methods:**

The study was conducted from October to December 2024. A total of 771 nurses completed a survey including the nine‐item Utrecht Work Engagement Scale (UWES‐9), the work ability index (WAI) and single‐item measures assessing job satisfaction and turnover intentions. One‐way ANOVA, chi‐square tests and GLM univariate multivariable analyses with Bonferroni‐corrected pairwise comparisons were conducted.

**Results:**

Nurses reported high levels of work engagement and job satisfaction, with no statistically significant differences in the overall work engagement score across generations. Unadjusted analyses indicated significant generational differences in WAI scores, UWES ‘absorption’ and internal turnover intention. However, after controlling for confounders, these differences were no longer statistically significant. A significant adjusted generational effect emerged for ‘vigour’, with generation *X* scoring significantly higher than Millennials.

**Conclusion:**

This study contributes to expanding knowledge of how nurses’ work engagement, work ability, job satisfaction and turnover intentions vary across generations. However, generational differences should be considered with caution as they may be affected by experiential variables. On the other hand, the adjusted effect on ‘vigour’ suggests that motivational resources differ across generations, with implications for strategies to sustain younger nurses’ engagement and retention.

**Patient or Public Contribution:**

No patient or public contribution.

**Reporting Method:**

STROBE guidelines for cross‐sectional studies.

## 1. Introduction

Within the healthcare system, nurses are the largest group of healthcare workers. As frontline healthcare providers, they are uniquely positioned within healthcare organisations and are key to ensuring the quality of care, as well as cultivating and maintaining positive work environments. However, the nursing profession continues to face significant challenges related to workforce stability and retention, which exacerbate adverse working conditions, care outcomes and patient safety [1]. The nursing shortage has reached a critical level worldwide, significantly impacting the delivery of appropriate nursing care. Globally, a substantial nursing shortage of 5.8 million nurses remained in 2023, with a projected slight decline to 4.1 million by 2030 [2]. In Italy, approximately 10,000 nurses leave the profession annually due to turnover and the profession’s limited attractiveness, further exacerbating the shortage, particularly evident in preventive and district‐level care settings, as well as in various specific nursing fields [3].

In recent years, healthcare policymakers have closely monitored nurses’ turnover rate. Frequent turnover in hospitals results in an increased workload, exacerbating nursing shortages. This exacerbated workload increases the risk of adverse events and creates a stressful environment that further contributes to the high turnover rate among nurses. Moreover, nurse turnover significantly reduces staffing levels, which adversely affects the quality of patient care and compromises patient safety [4], highlighting the importance of understanding factors associated with nurses’ retention.

Nurse turnover has been extensively explored in research; nonetheless, significant discrepancies remain when comparing actual nurse turnover with nurses’ turnover intention, which is a conscious consideration of leaving their current position. Turnover intention serves as an important predictor and early indicator of potential workforce changes, but it does not always directly lead to actual turnover. This gap highlights the complexity of nurses’ decisions to leave their jobs and the multifaceted nature of turnover, which encompasses demographic, organisational and job satisfaction determinants [5]. Demographic characteristics, such as younger age, fewer years of experience and male gender, have been linked to higher turnover rates as well as to organisational factors involving aspects of the work environment, including job status, workload and management support [5]. Job satisfaction is a crucial factor that is strongly and negatively linked to nurses’ intention to leave the organisation, serving as the primary determinant for their job retention [6].

Alongside these established factors influencing nurse turnover, recent evidence highlights the crucial role of perceived work ability and work engagement. An engaged work team contributes to better outcomes at both individual and organisational levels and can also improve nursing students’ clinical learning experience, leading to enhanced organisational attractiveness and retention in the long term [7].

Work engagement is described as a positive and fulfilling state characterised by ‘vigour’ (energy and resilience in meeting job demands), ‘dedication’ (a strong sense of significance, enthusiasm, inspiration, pride and challenge in one’s work) and ‘absorption’ (being deeply absorbed in work tasks, with full concentration and immersion) [8]. Research shows an inverse correlation between work engagement and turnover intention, suggesting that employees with low engagement tend to have a higher intention to leave their jobs [9]. Furthermore, work engagement can impact individuals’ work ability, defined as an employee’s capacity to meet the demands of the work environment by utilising both physical and psychological resources, including health, skills, attitudes and values [10]. Recent evidence also suggests that a significant correlation exists between work ability, job satisfaction, turnover intention [11, 12] and work engagement [12]. Specifically, work ability has a direct impact on job satisfaction and turnover intention. Moreover, job satisfaction influences nurses’ turnover intentions, regardless of their perceived work ability, and is considered a full mediator of the relationship between work ability and turnover intention [11]. Interestingly, the relationships between all the above variables differ between nurses younger than 45 years and those aged 45 years or older [12]. This variation suggests that generational differences may play a crucial role in shaping how nurses engage with their work, experience satisfaction and perceive their capacity to meet job demands, which in turn influence their turnover intentions and likelihood of remaining in their roles.

In this context, generational patterns within the nursing workforce add complexity to the dynamics of nurse turnover. Nurses’ organisational behaviours differ across generations, and these differences cannot be solely attributed to age; instead, they reflect distinct needs and values that influence their workplace attitudes and decisions.

The current nursing workforce comprises multiple generational cohorts, including Baby Boomers (BB), Generation *X*, Millennials (also known as Generation Y) and Generation *Z*, each characterised by distinct values, work ethics, attitudes and expectations [13]. For instance, BB, typically older and nearing retirement, generally value organisational loyalty and a strong work ethic, often preferring hierarchical organisational structures. In contrast, Generation *X* places a greater emphasis on independence and achieving autonomy, preferring task‐oriented activities. Millennials are characterised as innovation‐oriented and favouring change over stability, often seeking meaningful work that emphasises technological integration and workplace flexibility. Generation *Z* is the first generation to grow up fully immersed in digital technology and social media, and prioritises values such as workplace flexibility and work–life balance [14].

Although recent studies have investigated turnover intentions and job satisfaction within specific nursing generations, such as Generation *Z* [15–17], Generations Y and *Z* [18], and Generations *X*, *Y* and *Z* [19, 20], or examined work engagement, turnover intention and job satisfaction among BB, Generation *X* and *Y* nurses [21], current research focuses on a limited set of variables or generational cohorts.

Despite these insights, to date, no comprehensive research has simultaneously explored the generational differences in internal and external turnover intentions, work engagement, work ability and job satisfaction within a single study considering all four main nursing generational cohorts (BB, Generation *X*, Millennials and Generation *Z*).

## 2. The Study

### 2.1. Aim

This study aimed to examine generational patterns in work engagement, work ability, job satisfaction and internal and external turnover intentions among nurses in a hospital and community healthcare setting and to examine whether those patterns persist after controlling for sociodemographic and occupational variables.

## 3. Methods

### 3.1. Design, Setting and Sample Size Calculation

A cross‐sectional study was conducted from October to December 2024 in a healthcare facility that included both hospital and community settings, in a northern Italian Region.

### 3.2. Sampling, Inclusion Criteria and Sample Size Determination

A convenience sampling technique was used to recruit the eligible nurses. The study included all registered nurses directly involved in patient care in hospital or community settings, while nurses who had administrative, organisational or educational roles were excluded.

A one‐factor ANOVA was conducted to detect a statistical difference between groups. By assuming an effect size of 0.25, a type I error of 0.05 (alpha) and a power of 0.80, a sample of 180 nurses was required. Therefore, considering an expected refusal rate of 20%, 216 nurses were expected to participate. G∗Power V3.1 software was used to determine the sample size.

### 3.3. Participants

A total of 771 nurses out of 1002 completed the questionnaires (response rate: 77%). Participants’ mean age was 45.11 years (*SD* = 11.03), ranging from 23 to 67 years. The majority were female (*n* = 656/754, 87%), held undergraduate degrees (*n* = 577/745, 77%), and 60% (*n* = 466/771) worked in hospital settings. Nurses reported an average of 21.54 years (*SD* = 12.00) of nursing experience and 9.94 years (*SD* = 9.58) in their current ward. The full description of the sample characteristics is shown in Table 1.

**TABLE 1:** Sample description.

Characteristics	*N*	%	Mean	*SD*	Min	Max
Age	713		45.11	11.03	23	67
Years in nursing	731		21.54	12.00	0	46
Years in current unit	719		9.94	9.58	0	45
Working setting						
Hospital	466	60.4				
Community	305	39.6				
Gender						
Male	98	13.0				
Female	656	87.0				
Marital status						
Married/cohabiting	404	58.8				
Single/separated/widow	283	41.2				
Education						
Undergraduate	577	77.4				
Postgraduate/advanced	168	22.6				
Generations						
Baby Boomers	24	3.4				
Generation X	386	54.1				
Millennials	239	33.5				
Generation Z	64	9.0				

*Note:* The number of valid cases varies across variables due to missing data. %: percentages are calculated based on valid cases for categorical variables.

### 3.4. Data Collection Procedures and Measures

A paper‐based survey, including sociodemographic questions and validated instruments to assess nurses’ work ability, engagement, job satisfaction and turnover intentions, was distributed in the healthcare organisation, with the involvement of nursing coordinators. Additionally, participants were provided with an information letter detailing the study’s aims, procedures, confidentiality and data privacy information. Upon completion, participants anonymously returned the completed questionnaire in a sealed envelope to the nursing coordinators.

#### 3.4.1. Sociodemographic Variables, Job Satisfaction and Turnover Intentions

The following sociodemographic information was collected: participants’ age, gender, marital status, work setting (hospital, community), years in the nursing profession, years in the current ward/unit and educational level.

A single‐item measure using a Likert scale from 1 (minimum) to 7 (maximum) was used to assess (i) job satisfaction (‘I am satisfied with my current job’); (ii) internal turnover intention (‘I would like to move to another ward/service within this health care facility within 1 year’) and (iii) external turnover intention (‘I would like to move to another health care facility within 1 year’) [12].

#### 3.4.2. Nine‐Item Utrecht Work Engagement Scale (UWES‐9)

To assess nurses’ work engagement, the UWES‐9 [8] was used. The scale includes the following three subscales: ‘vigour’ (3 items), ‘dedication’ (3 items) and ‘absorption’ (3 items). Each item is rated on a 7‐point Likert scale, from 1 (*never*) to 7 (*always*). In this study, the overall Cronbach’s alpha for UWES was 0.91, with subscale scores of 0.89 for ‘vigour’, 0.82 for ‘dedication’ and 0.78 for ‘absorption’.

#### 3.4.3. Work Ability Index (WAI)

To assess the perceived work ability, the WAI [10] was used. It includes seven items to evaluate the mental and physical demands of the workplace and the individual’s health status: (1) estimation of current work ability compared with the best lifetime performance (0–10 points); (2) work ability relative to the demands of the job (2–10 points); (3) number of diseases diagnosed by a physician (1–7 points); (4) estimation of work impairment due to diseases (1–6 points); (5) sickness absenteeism in the past 12 months (1–5 points); (6) personal prognosis of work ability over the next two years (1, 4 or 7 points) and (7) personal mental resources as perceived in one’s own life (1–4 points).

The WAI total score is calculated by summing the item scores and ranges from 7 to 49 points. A higher WAI score indicates better work ability, categorised as: Category 1: ‘low’ (7–27 points); Category 2: ‘moderate’ (28–36 points); Category 3: ‘good’ (37–43 points) and Category 4: ‘excellent’ (44–49 points). In this study, the overall WAI Cronbach’s alpha was 0.82.

### 3.5. Data Analysis

Descriptive statistics were used to describe the sample and scale scores. Continuous variables were summarised using means and standard deviations (SDs), along with their minimum and maximum values. Categorical variables were reported as frequencies and percentages. Chi‐square tests and one‐way ANOVA assessed the statistical significance of differences between generational groups, which included the cohorts: BB (61–79 years), Generation *X* (45–60 years), Millennials (29–44 years) and Generation *Z* (13–28 years) [13]. Furthermore, subgroup analyses (*t*‐test and chi‐square) were performed to explore the differences between the outcome variables and the sociodemographic characteristics. Additionally, a series of multivariable analyses were conducted using general linear modelling (GLM) to test whether the generational differences were robust to the influence of potential confounders. Covariates included sociodemographic variables (i.e., gender, marital status, educational level, years in the nursing profession and years in the current ward/unit), and contextual factors, such as hospital or community work setting. Separate models were estimated for each outcome variable (WAI total score, UWES total score and its factors, job satisfaction and both internal and external turnover intentions) with generational cohort entered as a fixed factor and the sociodemographic and contextual variables as covariates.

Data were analysed using SPSS Version 29. Statistical significance was set at *p* < 0.05.

To evaluate the reliability of the instruments, Cronbach’s alpha was calculated. Values > 0.90 are considered excellent, > 0.70 and < 0.90 good, > 0.60 and < 0.70 acceptable, and < 0.60 nonacceptable [22].

### 3.6. Ethical Considerations

Ethical approval for this study was granted by Northumbria University (ref: 7338, approval date: 20 May 2024). Additionally, administrative permission was secured from the healthcare facility managers of the participating centres. Participation was entirely voluntary, based on informed consent, with data confidentiality and anonymity ensured. Data collection and analysis were conducted to protect confidentiality and adhered to National and European laws, including the Personal Data Act. Eligible participants received written information about the study’s aims, procedures, data management and ethical considerations. All completed questionnaires were returned in sealed envelopes. As stated in the information sheet, return of the questionnaire was considered as consent to participate in the study.

## 4. Results

### 4.1. Scales’ Descriptive Statistics

The descriptive statistics of the scales are shown in Table 2.

**TABLE 2:** Descriptive statistics of the scales.

Scales	*N*	Mean	*SD*	Min	Max
Work engagement (UWES)	753	5.20	1.15	1	7
Vigour	752	4.91	1.28	1	7
Dedication	752	5.40	1.32	1	7
Absorption	751	5.30	1.21	1	7
Work ability (WAI)	771	37.52	6.11	11	49
Job satisfaction	755	5.01	1.58	1	7
Internal turnover	749	2.64	2.17	1	7
External turnover	750	2.33	1.96	1	7

*Note:* The number of valid cases varies across scales due to missing data.

Abbreviations: UWES, Utrecht Work Engagement Scale; WAI, work ability index.

The mean total score on the UWES was 5.20 (*SD* = 1.15), indicating moderate to high levels of engagement. Among the UWES subscales, “dedication” had the highest mean score (*M* = 5.40, *SD* = 1.32), followed by “absorption” (*M* = 5.30, *SD* = 1.21) and “vigour” (*M* = 4.91, *SD* = 1.28).

Nurses’ work ability was rated as ‘good’, with a mean WAI total score of 37.52 (*SD* = 6.11). Job satisfaction had a mean score of 5.01 (*SD* = 1.58), while turnover intentions were relatively low for both internal turnover (*M* = 2.64, *SD* = 2.17) and external turnover (*M* = 2.33, *SD* = 1.96). Participants were categorised into four generational groups: BB (3%), Generation *X* (54%), Millennials (33.5%) and Generation *Z* (9%) (Table 3).

**TABLE 3:** Scales scores comparison between generations (ANOVA).

Scales	Generation	*N*	Mean	*SD*	*F*	*p*
Work engagement (UWES)	Baby Boomers	23	5.51	1.15	1.042	0.37
	Generation X	375	5.26	1.19		
	Millennials	236	5.16	1.04		
	Generation Z	64	5.14	1.15		

Vigour	Baby Boomers	23	5.38	1.20	2.097	0.10
	Generation X	374	5.00	1.32		
	Millennials	236	4.86	1.21		
	Generation Z	64	4.73	1.19		

Dedication	Baby Boomers	23	5.55	1.39	0.159	0.92
	Generation X	374	5.42	1.35		
	Millennials	236	5.43	1.20		
	Generation Z	64	5.51	1.35		

Absorption	Baby Boomers	23	5.61	1.04	2.973	0.03
	Generation X	373	5.43	1.18		
	Millennials	236	5.18	1.15		
	Generation Z	64	5.18	1.26		

Work ability (WAI)	Baby Boomers	24	36.17	5.64	7.581	< 0.001
	Generation X	386	36.90	6.20		
	Millennials	239	38.81	5.29		
	Generation Z	64	39.34	5.41		

Job satisfaction	Baby Boomers	23	5.61	1.70	1.192	0.31
	Generation X	381	5.09	1.52		
	Millennials	237	4.99	1.55		
	Generation Z	63	5.05	1.57		

Internal turnover	Baby Boomers	23	1.26	0.86	5.675	< 0.001
	Generation X	375	2.46	2.10		
	Millennials Y	238	2.79	2.17		
	Generation Z	63	3.16	2.42		
External turnover	Baby Boomers	23	1.78	1.76	0.597	0.62
	Generation X	376	2.28	1.93		
	Millennials Y	238	2.23	1.86		
	Generation Z	63	2.38	2.03		

*Note:* The number of valid cases varies across variables due to missing data.

Abbreviations: UWES, Utrecht Work Engagement Scale; WAI: work ability index.

### 4.2. Generational Differences in Work Engagement, Work Ability, Job Satisfaction and Turnover Intentions

#### 4.2.1. Work Engagement

No statistically significant differences across generations emerged in the UWES overall score (*F* = 1.04, *p* = 0.37), in the ‘vigour’ (*F* = 2.10, *p* = 0.10) and ‘dedication’ (*F* = 0.16, *p* = 0.92) subscales, while ‘absorption’ showed a significant difference (*F* = 2.97, *p* = 0.03), with BB reporting the highest absorption score (*M* = 5.61, *SD* = 1.04) compared to the other groups.

#### 4.2.2. WAI

Millennials (*M* = 38.81, *SD* = 5.29) and Generation *Z* (*M* = 39.34, *SD* = 5.41) scored significantly higher than BB (*M* = 36.17, *SD* = 5.64) and Generation *X* (*M* = 36.90, *SD* = 6.20) showing significant generational differences (*F* = 7.58, *p* < 0.001).

#### 4.2.3. Job Satisfaction

No significant differences in job satisfaction were observed across generations (*F* = 1.19, *p* = 0.31).

#### 4.2.4. Turnover Intentions

BB showed the lowest levels of both internal and external turnover intentions (*M* = 1.26, *SD* = 0.86; *M* = 1.78, *SD* = 1.76, respectively), while Generation *Z* showed the highest levels (internal turnover: *M* = 3.16, *SD* = 2.42; external turnover: *M* = 2.38, *SD* = 2.03).

Internal turnover intention differed significantly across generations (*F* = 5.68, *p* < 0.001). No statistically significant differences were observed among generations for external turnover intention (*F* = 0.60, *p* = 0.62).

### 4.3. WAI Categories by Generations and Work Setting

A significant association was found between generational cohort and WAI categories, *χ* ^2^ (9*, N* = 713) = 30.83, *p* < 0.001. BB (*n* = 24) were absent from the ‘low’ category and were most represented in the ‘moderate’ (50%) and ‘good’ (42%) categories. Generation *X* (*n* = 386) displayed a more distributed profile across all categories, with 8% in the lowest category, 35% in the ‘moderate’ category, 42% in the ‘good’ category and 15.5% in the ‘excellent’ category. Millennials (*n* = 239) and Generation *Z* (*n* = 64) participants were primarily represented in the higher WAI categories, with 57.3% of Millennials and 53.1% of Generation *Z* in the ‘good’ category, and 18% of Millennials and 22% of Generation *Z* in the ‘excellent’ category (Table 4).

**TABLE 4:** WAI categories by generations (chi‐square).

Generations	WAI categories									
	Low		Moderate		Good		Excellent		Total	
	*n*	%	*n*	%	*n*	%	*n*	%	*n*	%
Baby Boomers	—	—	12	50.0	10	41.7	2	8.4	24	100
Generation X	31	8.0	134	34.7	161	41.7	60	15.5	386	100
Millennials	12	5.0	48	20.1	137	57.3	42	17.6	239	100
Generation Z	3	4.7	13	20.3	34	53.1	14	21.9	64	100
Total	46	6.5	207	29.0	342	48.0	118	16.5	713^∗^	100

*Note:* Chi‐square test: *χ* ^2^(9, *N* = 713) = 30.83, *p* < 0.001. Percentages are row percentages within each generation.

^∗^The overall total is not 771 because of missing data in the variable ‘age’ used to categorise the generations. WAI: work ability index.

No significant differences were observed in WAI category distributions between nurses who work in hospital or community settings, *χ* ^2^ (3*, N* = 771) = 3.87, *p* = 0.28. The majority of nurses working in both hospital and community settings fell into the WAI ‘good’ category (hospital: 48%, *n* = 223; community: 46%, *n* = 139) (Table 5).

**TABLE 5:** WAI categories by healthcare setting (chi‐square).

Setting	WAI categories									
	Low		Moderate		Good		Excellent		Total	
	*n*	%	*n*	%	*n*	%	*n*	%	*n*	%
Hospital	31	6.7	144	30.9	223	47.9	68	14.6	466	100
Community	25	8.2	83	27.2	139	45.6	58	19.0	305	100
Total	56	7.3	227	29.4	362	47.0	126	16.3	771	100

*Note:* Chi‐square test: *χ* ^2^(3, *N* = 771) = 3.87, *p* = 0.28. Percentages are row percentages within each setting.

Abbreviation: WAI, work ability Iindex.

### 4.4. Multivariable and Subgroup Analyses

The subgroup analyses revealed few statistically significant differences for gender, with male participants mainly distributed among the highest WAI categories; postgraduate education, with those with postgraduate degrees presenting higher external turnover intention; and work setting, with those working in a hospital setting demonstrating higher internal and external turnover intention (Supporting tables A and B).

In the multivariable analysis, after controlling for all covariates, the generational effects on WAI total score, UWES ‘absorption’ and internal turnover intention were no longer statistically significant. No significant adjusted generational effects were observed for the remaining outcomes (all *p* > 0.10). By contrast, a significant adjusted generational effect emerged for ‘Vigour’ (*p* = 0.022). Adjusted marginal means indicated a declining trend from BB (*M* = 5.59) and Generation *X* (*M* = 5.20) to Millennials (*M* = 4.63) and Generation *Z* (*M* = 4.42). Bonferroni‐corrected pairwise comparisons identified a significant difference between Generation *X* and Millennials (*p* = 0.029); no other pair reached significance after correction. Across models, years of nursing experience demonstrated statistical significance for WAI (*p* < 0.001), total work engagement (*p* = 0.012), ‘vigor’ (*p* = 0.002) and ‘dedication’ (*p* = 0.013). Years in current ward/unit were independently significant for five outcomes, and work setting was a significant factor associated with ‘dedication’ and with internal turnover intention. Gender, educational level and marital status showed no consistent independent effects across models (Supporting tables C and D).

## 5. Discussion

This study examined the dynamics among nurses’ generational groups in terms of work engagement, perceived work ability, job satisfaction and turnover intentions. The results revealed that nurses’ work engagement and job satisfaction scored consistently high across generations, with no statistically significant differences. The overall turnover intentions were relatively low; nonetheless, internal turnover intentions exhibited variation across generations. These results highlight a counterintuitive pattern: Although Millennials and Generation *Z* reported higher scores in work ability, a well‐known protective factor against turnover intentions [23, 24], they also reported higher turnover intentions. However, recent studies suggested that WAI does not have a direct impact on nurses’ intentions to leave [11] and that there is no significant association between work ability and intention to leave the nursing profession [25]. Significant generational differences in WAI scores were observed, indicating variation in nurses’ perceived work ability across generations. However, this statistically significant difference aligns with expectations, and it is mainly related to ageing rather than a specific generational characteristic, confirming established patterns in the literature that considers the known age‐related differences in physical and mental demands [26]. Where generational differences were observed in unadjusted analyses, multivariable models were used to assess their robustness to sociodemographic and occupational confounders, with results indicating that, alongside some generational aspects, years of nursing experience should be considered as a relevant variable affecting several outcome measures. Moreover, the multivariable analyses partially mitigated the unbalanced distribution of the sample across the generations, as the BB and Generation *Z* represent two smaller proportions of the sample. While this sample distribution is representative of the actual age distribution of the Italian nursing workforce at the national level [3], the analytical approach further contributed both to controlling for confounders and mitigating this aspect.

### 5.1. Work Engagement Among Nursing Generations

The overall UWES scores and all the subscales’ scores (‘vigor’, ‘dedication’ and ‘absorption’) scored high and did not differ significantly across generations. However, it is interesting to consider the UWES subscale scores in the unadjusted analyses, and, in particular, note that ‘absorption’ was the only factor showing significant variation. Specifically, BB reported higher levels of ‘absorption’ compared to younger cohorts. Older nurses, with more years of experience and greater professional expertise, could be more likely to be entirely focused on their work, expressing a stronger professional identity, occupational stability, intrinsic motivation and ability to handle demanding roles. They may be more motivated by prosocial factors, such as teamwork and cohesiveness [27], as well as autonomy and factors related to task requirements [27], and organisational loyalty, reflecting traditional work values. These results contribute to a broader understanding of these complex dynamics within nurses’ work teams. Specifically, they expand the existing literature on this topic, which has shown that psychologically engaged employees exhibit more helping behaviours towards colleagues (consistent with the values of the BB generation) and are less likely to express turnover intentions.

In contrast, younger generations expressed lower ‘absorption’ scores, which could indicate that they may experience work engagement through different dimensions, such as ‘vigour’ or ‘dedication’. These dimensions are shaped by motivational priorities like work–life balance, autonomy and structured support from nurse managers [27, 28], aligning with these generational characteristics [28]. Previous research highlighted a statistically significant positive correlation between younger nurses’ generations and the need for work–life balance [28], an established factor influencing younger nurse retention [29], as well as professional career opportunities [12]. Therefore, the results of this study emphasise the significance of viewing work engagement as a multidimensional construct affected by the interaction of different generational, sociodemographic, professional and organisational variables. This might indicate varying underlying motivations for work engagement across generations. In this vein, multivariable analyses revealed that this pattern changed after controlling for covariates, and especially years in the nursing profession, suggesting that work engagement and ‘absorption’ may reflect professional expertise and role stability rather than a generational characteristic alone. Moreover, a significant adjusted generational effect emerged for ‘vigour’, with Generation *X* scoring significantly higher than Millennials after Bonferroni correction, independent of experience and all other covariates. It is also interesting to note that a previous similar study conducted in Italy revealed that ‘absorption’ did not significantly contribute to work ability, while ‘vigor’ and ‘dedication’ significantly contributed to it across different age groups [12]. Considering this evidence, it could be hypothesised that nurses from different generational cohorts exhibit work engagement differently, likely due to a combination of generational characteristics and age‐specific aspects linked to the professional experience.

Successfully engaging intergenerational groups in the workplace requires nurse managers to understand each generation’s distinct values, career stages and expectations to effectively enhance their respective strengths [28].

### 5.2. Nurses’ Work Ability Profiles Across Generations

The statistically significant difference in WAI by generation aligns with previous evidence and confirms that these differences are mainly driven by age‐related factors rather than a generational pattern. Significant generational differences emerged in the distribution of WAI categories, with younger nurses (Millennials and Generation Z) more frequently classified into higher work ability categories than older nurses (BB and Generation X). This pattern aligns with previous evidence [30, 31], suggesting a natural decline in physical and mental abilities with age. It should also be considered that older nurses face various work‐related challenges, including workload, alongside the physical effects of ageing. While multivariable models further confirmed that years of nursing experience (highly correlated with age) was the major factor affecting WAI, understanding the generational patterns in the nursing workforce can help healthcare managers target interventions to optimise nurses’ functional capacity throughout their careers, by considering both the sociodemographic components and the generational characteristics of the workforce. In addition, the consideration of the inter‐related nature of these dynamics supports informed decision‐making on workforce planning, work culture and staff retention, thereby improving workforce sustainability across all care settings [32].

Regarding the association between work settings and WAI categories, results showed that nurses’ work ability does not differ significantly between those working in hospital environments and those in community settings. This may indicate that elements affecting work ability are more closely related to different variables than just the workplace setting, such as individual‐level factors [33]. In particular, this study highlighted a statistically significant association between gender and work ability, where male nurses tend to demonstrate higher scores.

### 5.3. Generational Differences in Job Satisfaction and Turnover Intentions

This study revealed high levels of job satisfaction among nurses, with no statistically significant differences across generations. However, nurses in Generations Y and *Z* reported lower job satisfaction than those in the BB and Generation *X* cohorts. This is consistent with previous evidence, which showed that older nurses reported higher satisfaction levels than their younger colleagues [34]. Notably, younger generations, who expressed lower levels of job satisfaction, also reported lower overall UWES scores (total and subscales) and higher turnover intentions, while showing higher work ability. It is important to note that literature has extensively demonstrated that job satisfaction affects nurses’ retention [35, 36]. However, job satisfaction is a complex and multifaceted concept, and it relates not only to an individual’s perception of their job but also to the nature of the role and personal expectations of the work environment. A recent study found that job satisfaction mediated the relationship between WAI, turnover intention and the intent to leave the nursing profession and independently affected nurses’ organisational turnover intention [11]. Therefore, developing strategies to improve job satisfaction and retention is a priority for nurse managers, particularly among young nurses, to foster work engagement and reduce turnover [37]. In fact, this study revealed a significant difference between generations, particularly when considering internal turnover intentions. Younger nurses, especially those from Generation *Z*, showed the highest turnover intentions within the same healthcare facility. Conversely, BB exhibited the lowest levels of both internal and external turnover intentions. Although this finding may not be entirely affected by generations in the multivariable analysis, it aligns with existing literature, which indicates that demographic factors, such as younger age, are often associated with higher turnover rates [5, 20]. These differences in internal turnover intentions can be understood in the context of each cohort’s distinct expectations and priorities. As previously mentioned, younger nurses tend to value workplace flexibility, technological integration, professional growth and a healthy work–life balance [28]. Therefore, their increased internal turnover intention may reflect a proactive search for roles that better align with these values, offering diverse experiences, career development, new learning opportunities or improved work–life integration within the same organisation. While this generational difference disappeared in the multivariable models, suggesting that younger nurses’ higher internal mobility intentions may reflect career stage and limited institutional embeddedness rather than a generational value system, the generational lenses are theoretically consistent and should be taken into consideration alongside other sociodemographic and organisational variables. In contrast, BB often prioritise organisational loyalty and a strong work ethic, preferring stability and hierarchical structures [13]. Their lower turnover intentions, both internal and external, may stem from a stronger sense of professional identity developed over years of experience, along with a preference for maintaining their current roles, which may be associated with the higher absorption scores observed in the unadjusted analyses. While these considerations are consistent with established theoretical frameworks and previous research regarding generational patterns and characteristics, it is also necessary to consider the interaction between generations and age and its impact on work engagement, job satisfaction and turnover intentions.

### 5.4. Limitations and Strengths

This study has some limitations that should be recognised. Firstly, its cross‐sectional design limits the identification of causal relationships between generational differences, work engagement, work ability, job satisfaction and turnover intentions. Moreover, the observed generational differences may reflect a combination of generational values, age‐related changes, career stage, health status and proximity to retirement. Secondly, the study was conducted at a single centre in northern Italy, which may limit the generalisability of the results to other Italian regions or countries with different healthcare organisational contexts. Thirdly, data were collected through self‐reported questionnaires, which can introduce response bias and social desirability effects. Another limitation of this study is the potential bias introduced by the sample composition, which primarily consisted of nurses from Generations *X* and Y, with Generation *Z* and BB underrepresented. While this distribution properly represents the age distribution of the nursing workforce at the national level [3], this may also have affected the detection of statistically significant differences among generational groups.

Despite the above limitations, this study achieved a very high response rate, which strengthens the reliability and validity of these results, and reproduced the generational composition of the nursing workforce at the national level. Moreover, the large sample size, alongside the high response rate, is a major aspect mitigating the response bias and social desirability bias. Furthermore, including all active nursing generations offers a comprehensive overview of generational differences within the workforce, providing a valuable contribution to understanding nursing workforce dynamics and tailoring human resource management strategies. The multivariable analytical approach further strengthened the generational comparison by addressing the confounding variables.

### 5.5. Implications for Nursing Management and Future Research

Based on this study, nurse managers and healthcare organisations can better understand workplace dynamics and tailor interventions to the diverse needs of nurses across generations. This study reframes nursing workforce management from generational to experiential, while still revealing a generational effect. Managers should combine experience‐based and generationally sensitive strategies rather than relying on age or generational cohort alone.

Specifically, nurse managers should consider: (1) sustaining younger nurses’ vigour and engagement by protecting time for focused work, providing regular feedback and recognition and designing roles that foster absorption; (2) supporting older nurses’ work capacity through workload adjustments, ergonomic and workplace adaptations, and flexible scheduling that accommodate age‐related changes and physical demands; (3) managing internal mobility and offering timely rotation, and role redesign that foster dedication and retain expertise within the organisation; (4) tailoring career development to career stage, providing early‐career nurses with structured mentoring and clear advancement pathways while offering experienced nurses roles in mentoring, education, and clinical leadership; and (5) strengthening intergenerational teams through cross‐generational mentoring, knowledge‐sharing and team‐building that leverage the complementary strengths of each generational cohort. Together, these experientially and generationally sensitive approaches allow managers to address specific challenges and strengths within their teams, fostering a supportive environment that promotes well‐being, job satisfaction and sustained workforce stability.

From a research perspective, future multicentre studies are needed and should include a more balanced representation of different generations of nurses to improve the generalisability of results and reduce sampling bias. In addition, longitudinal studies may provide more robust evidence and a deeper understanding of how nurses’ work engagement, work ability, job satisfaction and turnover intentions evolve and change throughout their careers. The interaction between generational values, age‐related changes, career stage and other sociodemographic variables, while addressed by supporting multivariable statistics and subgroup analyses, should be further explored with longitudinal and cohort sequential designs capable of disentangling these variables. This would make it possible to address the age–period–cohort (APC) problem, widely known in generational research, according to which these generational and age patterns are multicollinear, making it statistically challenging, without additional assumptions or adequate study designs, to separate their independent effects [38]. Finally, employing mixed‐methods approaches that integrate both quantitative and qualitative data can more effectively capture the motivational drivers of engagement across different generational cohorts.

## 6. Conclusion

This study offers valuable insights into generational differences in nurses’ work engagement, work ability, job satisfaction and turnover intentions. While unadjusted analyses suggested significant generational differences in work ability, work absorption and internal turnover intention, multivariable analyses revealed that these differences were attributable to years of nursing experience rather than to generational characteristics alone. This finding reframes the narrative from generational management to experience management, suggesting that cumulative occupational demands, rather than generation‐specific values alone, are relevant drivers of nurses’ work outcomes. However, a significant independent generational effect emerged for ‘vigour’ after covariate adjustment, with Generation *X* reporting higher energy and resilience than Millennials, indicating that motivational resources may genuinely differ across generations in ways that are not fully explained by career stage or experience. These results call for nurse leaders to invest in strategies that consider both experiential and generational factors to sustain younger nurses’ work engagement and address the occupational demands that accumulate with experience. Taken together, these findings suggest that the combination of sociodemographic, professional and generational differences can significantly influence the relationship between work ability and turnover intentions, involving new challenges and opportunities for nursing leaders in developing personalised strategies that effectively engage and retain the next generation of nurses.

## Author Contributions

Dania Comparcini: writing–original draft preparation and writing–reviewing and editing. Eleonora Paro: conceptualisation, methodology, writing–original draft preparation, supervision and writing–reviewing and editing. Giuseppe Tonutti: writing–reviewing and editing, supervision and conceptualisation. Michele Chittaro: conceptualisation and writing–reviewing and editing. Mariagrazia Valentini and Rosanna Finos: visualisation and writing–reviewing and editing. Marco Tomietto: conceptualisation, methodology, software, formal analysis, data curation, writing–original draft preparation, supervision, validation and writing–reviewing and editing.

## Funding

This research received no specific grant from any funding agency in the public, commercial or not‐for‐profit sectors.

## Disclosure

Implications for Nursing Management: This study provides nurse managers with useful insights to plan tailored approaches to enhance staff retention, based on workforce characteristics. Offering targeted development opportunities for younger nurses and using cross‐generational mentoring can strengthen intergenerational team cohesion and improve organisational outcomes.

There is an author with advanced expertise in statistics—Professor Marco Tomietto.

## Ethics Statement

Ethical approval for this research was granted by the Institutional Review Board at Northumbria University (reference number: 7338; date of approval: 20 May 2024).

## Conflicts of Interest

The authors declare no conflicts of interest.

## Supporting Information

Additional supporting information can be found online in the Supporting Information section.

## Supporting information


**Supporting Information 1** Supporting table A. Scale scores comparison by sociodemographic variables (independent samples *t*‐tests). Supporting table B. WAI categories by sociodemographic variables (chi‐square). Supporting Table C. Covariate‐adjusted estimated marginal means by generational cohort and omnibus generation effects (GLM). Supporting Table D. Effects of sociodemographic and occupational covariates across GLM models: F statistics and significance.

**Supporting Information 2** STROBE_checklist_cross‐sectional.

## Data Availability

The data that support the findings of this study are available upon request from the corresponding author. The data are not publicly available due to privacy or ethical restrictions.

## References

[1] Bae S. H. , Noneconomic and Economic Impacts of Nurse Turnover in Hospitals: A Systematic Review, International Nursing Review. (2022) 69, no. 3, 392–404, 10.1111/inr.12769. 35654041 PMC9545246

[2] World Health Organization , State of the World’s Nursing 2025: Investing in Education, Jobs, Leadership and Service Delivery, 2025, https://www.who.int/publications/i/item/9789240110236.

[3] Federazione Nazionale Ordini Professioni Infermieristiche (FNOPI) , Rapporto Professioni Infermieristiche 2025: Professione Essistema in Evoluzione [Nursing Professions Report 2025: Profession and Evolving System], 2025, https://www.fnopi.it/wp-content/uploads/2025/05/00_Libro_FNOPI_2025_versioneWEB-ok.pdf.

[4] Antwi Y. A. and Bowblis R. , The Impact of Nurse Turnover on Quality of Care and Mortality in Nursing Homes: Evidence From the Great Recession, American Journal of Health Economics. (2018) 4, no. 2, 131–163, 10.1162/ajhe_a_00096.

[5] Wu F. , Lao Y. , Feng Y. , Zhu J. , Zhang Y. , and Li L. , Worldwide Prevalence and Associated Factors of Nursing Staff Turnover: A Systematic Review and Meta Analysis, Nursing Open. (2024) 11, no. 1, 10.1002/nop2.2097. PMC10802134 38268271

[6] De Vries N. , Lavreysen O. , Boone A. et al., Retaining Healthcare Workers: A Systematic Review of Strategies for Sustaining Power in the Workplace, Healthcare. (2023) 11, no. 13, 10.3390/healthcare11131887. PMC10341299 37444721

[7] Tomietto M. , Comparcini D. , Simonetti V. et al., Work-Engaged Nurses for a Better Clinical Learning Environment: A Ward-Level Analysis, Journal of Nursing Management. (2016) 24, no. 4, 475–482, 10.1111/jonm.12346. 26645780

[8] Bakker A. B. and Leiter M. P. , Work Engagement: A Handbook of Essential Theory and Research, 2010, Psychology Press, London, UK, 10.4324/9780203853047.

[9] Takawira N. , Coetzee M. , and Schreuder D. , Job Embeddedness, Work Engagement and Turnover Intention of Staff in a Higher Education Institution: An Exploratory Study, SA Journal of Human Resource Management. (2014) 12, 1–10, 10.4102/sajhrm.v12i1.524.

[10] Tuomi K. , Ilmarinen J. , Jahkola A. , Katajarinne L. , and Tulkki A. , Work Ability Index, 1998, 2nd edition, Finnish Institute of Occupational Health, Helsinki, 10.1093/occmed/kqm008.

[11] Comparcini D. , Simonetti V. , Totaro M. et al., Impact of Traumatic Stress on Nurses′ Work Ability, Job Satisfaction, Turnover, and Intention to Leave: A Cross-Sectional Study, Journal of Advanced Nursing. (2025) 81, no. 10, Advance online Publication, 6504–6514, 10.1111/jan.16796. 39930285

[12] Tomietto M. , Paro E. , Sartori R. , et al., PN Nursing Group , Work Engagement and Perceived Work Ability: An Evidence-Based Model to Enhance Nurses′ Well-Being, Journal of Advanced Nursing. (2019) 75, no. 9, 1933–1942, 10.1111/jan.13981. 30791116

[13] Parry E. and Urwin P. , Generational Differences in Work Values: A Review of Theory and Evidence, International Journal of Management Reviews. (2011) 13, no. 1, 79–96, 10.1111/j.1468-2370.2010.00285.x.

[14] Kanste O. , Ylisirniö M. , Hammarén M. , and Kuha S. , The Perceptions of Generation Z Professionals and Students Concerning Health-Care Work: A Scoping Review, Nurse Education Today. (2025) 150, 10.1016/j.nedt.2025.106678. 40090113

[15] Jeong K. Y. , Yun M. , and Choi E. H. , The Effects of Communication Competence, Meaning of Work, and Work-Life Balance on Turnover Intention in Generation Z Nurses in South Korea: A Cross-Sectional Study, Applied Nursing Research. (2025) 83, 10.1016/j.apnr.2025.151952. 40473354

[16] Lee Y. J. , Lee H. , and Choi E. H. , Moderating Role of Communication Competence in the Association Between Professionalism and Job Satisfaction in Korean Millennial and Generation Z Nurses: A Cross-Sectional Study, Healthcare. (2023) 11, no. 18, 10.3390/healthcare11182547. PMC10531258 37761744

[17] Rohayati T. , Destalani A. A. , Arizka H. D. , Fahrezi M. D. , and Dwidienawati D. , Impact of Job Satisfaction, Positive Organizational Culture and Meaningful Work on Turnover Intention in Gen Z, WSEAS Transactions on Systems. (2023) 22, 613–621, 10.37394/23202.2023.22.62.

[18] Kim E. , Kim H. , and Lee T. , How are New Nurses Satisfied With Their Jobs? From the Work Value Perspective of Generations Y and Z Nurses, BMC Nursing. (2024) 23, no. 1, 10.1186/s12912-024-01928-7. PMC11032593 38643129

[19] Lee S. A. and Lee J. , Differences in Occupational Values, Communication Types, Job Satisfaction, and Organisational Commitment Among Clinical Nurses Across Generations, Frontiers in Psychology. (2023) 14, 10.3389/fpsyg.2023.1174197. PMC10379635 37519366

[20] Hu G. , Wang Z. , Zhang C. et al., Intergenerational Differences in Turnover Intention of Nurses: A Cross-Sectional Survey in Jiangsu Province, China, Frontiers in Psychiatry. (2025) 16, 10.3389/fpsyt.2025.1550623. PMC12119595 40443754

[21] Sanches D. , Pereira S. , Castro S. , Mendes M. , Santos E. , and Ribeiro O. , Generational Diversity in Nursing Practice Environments–Scoping Review, BMC Nursing. (2024) 23, no. 1, 10.1186/s12912-024-02607-3. PMC11657357 39702311

[22] DeVellis R. F. , Scale Development: Theory and Applications, 2016, 4th edition, Sage Publications, https://collegepublishing.sagepub.com/products/scale-development-4-246123.

[23] Derycke H. , Clays E. , Vlerick P. , D′Hoore W. , Hasselhorn H. M. , and Braeckman L. , Perceived Work Ability and Turnover Intentions: A Prospective Study Among Belgian Healthcare Workers, Journal of Advanced Nursing. (2012) 68, no. 7, 1556–1566, 10.1111/j.1365-2648.2012.05961.x. 22348810

[24] Rongen A. , Robroek S. J. , van der Heijden B. I. , Schouteten R. , Hasselhorn H. M. , and Burdorf A. , Influence of Work-Related Characteristics and Work Ability on Changing Employer or Leaving the Profession Among Nursing Staff, Journal of Nursing Management. (2014) 22, no. 8, 1065–1075, 10.1111/jonm.12066. 23941401

[25] Čukljek S. , Babić J. , Ilić B. et al., Associations Between Quality of Nursing Work Life, Work Ability Index and Intention to Leave the Workplace and Profession: A Cross-Sectional Study Among Nurses in Croatia, International Journal of Environmental Research and Public Health. (2025) 22, no. 8. 10.3390/ijerph22081192 PMC12386502 40869778

[26] Paneni V. , Gambelunghe C. , Tomassini L. et al., Correlations Between Gender, Age, and Occupational Factors on the Work Ability Index Among Healthcare Professionals, Healthcare. (2025) 13, no. 7, 10.3390/healthcare13070702. PMC11988494 40218000

[27] Campbell C. M. and Patrician P. A. , Generational Preferences in the Nursing Work Environment: A Dimensional Concept Analysis, Journal of Nursing Management. (2020) 28, no. 4, 927–937, 10.1111/jonm.13024. 32255219

[28] Tan S. H. E. and Chin G. F. , Generational Effect on Nurses’ Work Values, Engagement, and Satisfaction in an Acute Hospital, BMC Nursing. (2023) 22, no. 1. 10.1186/s12912-023-01256-2 PMC10061355 36997911

[29] Kim E. Y. , Kim S. H. , and Oh Y. K. , Impact of Nurse Practice Environment, Coworker Support, and Work-Life Balance on Job Satisfaction for Newly Graduated Nurses, Korean Journal of Occupational Health Nursing. (2021) 30, no. 1, 1–9, 10.5807/kjohn.2021.30.1.1.

[30] Casolari L. , Curzi Y. , Mastroberardino M. et al., Factors Associated With Work Ability Among Employees of an Italian University Hospital, BMC Health Services Research. (2024) 24, no. 1, 10.1186/s12913-023-10465-z. PMC10768426 38178153

[31] Garzaro G. , Clari M. , Ciocan C. et al., Physical Health and Work Ability Among Healthcare Workers: A Cross-Sectional Study, Nursing Reports. (2022) 12, no. 2, 259–269, 10.3390/nursrep12020026. 35466246 PMC9036298

[32] Schüttengruber G. , Olsson M. M. , Holmberg C. et al., Understanding Ageism Towards Older Nursing Staff and Service Users: A Systematic Mapping Review From the Perspective of Clinical Leaders and Healthcare Managers, Geriatric Nursing. (2024) 58, 171–182, 10.1016/j.gerinurse.2024.05.017. 38820985

[33] Witkoski Stimpfel A. , Arabadjian M. , Liang E. , Sheikhzadeh A. , Schecter Weiner S. , and Vaughan Dickson V. , Organization of Work Factors Associated With Work Ability Among Aging Nurses, Western Journal of Nursing Research. (2020) 42, no. 6, 397–404, 10.1177/0193945919866218. 31322064 PMC6980255

[34] Halcomb E. , Smyth E. , and McInnes S. , Job Satisfaction and Career Intentions of Registered Nurses in Primary Health Care: An Integrative Review, BMC Family Practice. (2018) 19, no. 1, 10.1186/s12875-018-0819-1. PMC6081816 30086722

[35] O’Callaghan C. and Sadath A. , Exploring Job Satisfaction and Turnover Intentions Among Nurses: Insights From a Cross-Sectional Study, Cogent Psychology. (2025) 12, no. 1, 10.1080/23311908.2025.2481733.

[36] Edwards-Dandridge Y. , Simmons B. D. , and Campbell D. G. , Predictor of Turnover Intention of Registered Nurses: Job Satisfaction or Work Engagement?, International Journal of Applied Management and Technology. (2020) 19, no. 1, 87–96, 10.5590/IJAMT.2020.19.1.07.

[37] Wei H. , Horsley L. , Cao Y. et al., The Associations Among Nurse Work Engagement, Job Satisfaction, Quality of Care, and Intent to Leave: A National Survey in the United States, International Journal of Nursing Science. (2023) 10, no. 4, 476–484, 10.1016/j.ijnss.2023.09.010. PMC10667320 38020845

[38] Yang Y. and Land K. C. , Age-Period-Cohort Analysis: New Models, Methods, and Empirical Applications, 2013, Taylor & Francis, 10.1201/b13902.

