# Supplementary material for: Generational Patterns in Work Engagement, Work Ability, Job Satisfaction and Turnover Intentions Among Nurses: A Cross‐Sectional Study
Source: J Nurs Manag. 2026 Aug 19;2026:9713267. doi: 10.1155/jonm/9713267 (PMC13490706; doi:10.1155/jonm/9713267)
Supplement: Supplementary file 1 [file JONM-2026-9713267-s001.docx]

**Supplementary Table A**

*Scale scores comparison by sociodemographic variables (independent samples t-tests)*

| **Scales** | **Group** | ***Mean*** | ***SD*** | ***t*** | ***p*** |
| --- | --- | --- | --- | --- | --- |
|  | |  |  |  |  |
|  | **Gender** |  |  |  |  |
| Work Engagement (UWES) | Male | 5.31 | 1.09 | *0.87* | .383 |
|  | Female | 5.20 | 1.15 |  |  |
| Vigor | Male | 5.09 | 1.27 | *1.32* | .188 |
|  | Female | 4.90 | 1.28 |  |  |
| Dedication | Male | 5.56 | 1.17 | *1.10* | .270 |
|  | Female | 5.41 | 1.32 |  |  |
| Absorption | Male | 5.28 | 1.31 | *−0.35* | .724 |
|  | Female | 5.32 | 1.18 |  |  |
| Job Satisfaction | Male | 5.28 | 1.56 | *1.62* | .106 |
|  | Female | 5.00 | 1.56 |  |  |
| External Turnover Int. | Male | 2.37 | 1.98 | *0.33* | .738 |
|  | Female | 2.30 | 1.95 |  |  |
| Internal Turnover Int. | Male | 2.68 | 2.09 | *0.21* | .834 |
|  | Female | 2.63 | 2.18 |  |  |
|  | |  |  |  |  |
|  | **Education** |  |  |  |  |
| Work Engagement (UWES) | Undergraduate | 5.21 | 1.14 | *−0.17* | .869 |
|  | Postgraduate/Advanced | 5.22 | 1.13 |  |  |
| Vigor | Undergraduate | 4.92 | 1.27 | *−0.52* | .606 |
|  | Postgraduate/Advanced | 4.98 | 1.28 |  |  |
| Dedication | Undergraduate | 5.43 | 1.30 | *0.29* | .775 |
|  | Postgraduate/Advanced | 5.40 | 1.29 |  |  |
| Absorption | Undergraduate | 5.31 | 1.18 | *0.12* | .906 |
|  | Postgraduate/Advanced | 5.30 | 1.22 |  |  |
| Job Satisfaction | Undergraduate | 5.03 | 1.55 | *0.04* | .967 |
|  | Postgraduate/Advanced | 5.02 | 1.66 |  |  |
| External Turnover Int. | Undergraduate | 2.19 | 1.86 | *−2.89** | .004 |
|  | Postgraduate/Advanced | 2.72 | 2.14 |  |  |
| Internal Turnover Int. | Undergraduate | 2.61 | 2.15 | *−0.61* | .538 |
|  | Postgraduate/Advanced | 2.72 | 2.20 |  |  |
|  | |  |  |  |  |
|  | **Marital Status** |  |  |  |  |
| Work Engagement (UWES) | Married/Cohabiting | 5.22 | 1.19 | *−0.16* | .872 |
|  | Single/Separated/Widowed | 5.23 | 1.07 |  |  |
| Vigor | Married/Cohabiting | 4.97 | 1.33 | *0.58* | .566 |
|  | Single/Separated/Widowed | 4.92 | 1.20 |  |  |
| Dedication | Married/Cohabiting | 5.41 | 1.34 | *−0.39* | .700 |
|  | Single/Separated/Widowed | 5.45 | 1.26 |  |  |
| Absorption | Married/Cohabiting | 5.33 | 1.19 | *−0.08* | .935 |
|  | Single/Separated/Widowed | 5.34 | 1.17 |  |  |
| Job Satisfaction | Married/Cohabiting | 5.12 | 1.50 | *1.04* | .299 |
|  | Single/Separated/Widowed | 5.00 | 1.64 |  |  |
| External Turnover Int. | Married/Cohabiting | 2.20 | 1.89 | *−0.27* | .787 |
|  | Single/Separated/Widowed | 2.24 | 1.86 |  |  |
| Internal Turnover Int. | Married/Cohabiting | 2.42 | 2.05 | *−2.25** | .025 |
|  | Single/Separated/Widowed | 2.80 | 2.23 |  |  |
|  | |  |  |  |  |
|  | **Work Setting** |  |  |  |  |
| Work Engagement (UWES) | Hospital | 5.22 | 1.14 | *0.81* | .417 |
|  | Community | 5.15 | 1.17 |  |  |
| Vigor | Hospital | 4.91 | 1.27 | *−0.01* | .992 |
|  | Community | 4.91 | 1.30 |  |  |
| Dedication | Hospital | 5.47 | 1.30 | *1.77* | .078 |
|  | Community | 5.30 | 1.33 |  |  |
| Absorption | Hospital | 5.29 | 1.24 | *−0.38* | .703 |
|  | Community | 5.32 | 1.16 |  |  |
| Job Satisfaction | Hospital | 4.95 | 1.60 | *−1.26* | .206 |
|  | Community | 5.10 | 1.54 |  |  |
| External Turnover Int. | Hospital | 2.46 | 2.00 | *2.32** | .021 |
|  | Community | 2.12 | 1.88 |  |  |
| Internal Turnover Int. | Hospital | 2.87 | 2.24 | *3.69** | <.001 |
|  | Community | 2.29 | 2.01 |  |  |

**Supplementary Table B**

*WAI Categories by sociodemographic variables (Chi-Square)*

|  | **Low** | | **Moderate** | | **Good** | | **Excellent** | | **Total** | |
| --- | --- | --- | --- | --- | --- | --- | --- | --- | --- | --- |
|  | ***n*** | ***%*** | ***n*** | ***%*** | ***n*** | ***%*** | ***n*** | ***%*** |  |  |
| **Gender** | | | | | | | | | | |
| Male | 3 | 3.1 | 22 | 22.4 | 48 | 49.0 | 25 | 25.5 | 98 | 100 |
| Female | 49 | 7.5 | 195 | 29.7 | 312 | 47.6 | 100 | 15.2 | 656 | 100 |
| **Total** | 52 | 6.9 | 217 | 28.8 | 360 | 47.7 | 125 | 16.6 | **754** | 100 |
| *Chi-square test: χ²(3, N = 754) = 9.428, p = .024* | | | | | | | | | | |
| **Education** | | | | | | | | | | |
| Undergraduate | 38 | 6.6 | 172 | 29.8 | 270 | 46.8 | 97 | 16.8 | 577 | 100 |
| Postgraduate/Advanced | 11 | 6.5 | 45 | 26.8 | 85 | 50.6 | 27 | 16.1 | 168 | 100 |
| **Total** | 49 | 6.6 | 217 | 29.1 | 355 | 47.7 | 124 | 16.6 | **745** | 100 |
| *Chi-square test: χ²(3, N = 745) = 0.846, p = .838* | | | | | | | | | | |
| **Marital Status** | | | | | | | | | | |
| Married/Cohabiting | 27 | 6.7 | 125 | 30.9 | 193 | 47.8 | 59 | 14.6 | 404 | 100 |
| Single/Separated/Widowed | 13 | 4.6 | 73 | 25.8 | 142 | 50.2 | 55 | 19.4 | 283 | 100 |
| **Total** | 40 | 5.8 | 198 | 28.8 | 335 | 48.8 | 114 | 16.6 | **687** | 100 |
| *Chi-square test: χ²(3, N = 687) = 5.314, p = .150* | | | | | | | | | | |
|  | | | | | | | | | | |

**Supplementary Table C**

*Covariate-adjusted estimated marginal means by generational cohort and omnibus generation effects (GLM)*

| **Scale/Outcome** | **Baby Boomers**  *M (SE)* | **Generation X**  *M (SE)* | **Millennials** *M (SE)* | **Generation Z**  *M (SE)* | ***F*** | ***p*** | **η²p** |
| --- | --- | --- | --- | --- | --- | --- | --- |
| *Effect df = 3 for all models; error df varies (see note)* | | | | | | | |
| Work Ability Index (WAI) | 38.90 (1.44) | 38.47 (.47) | 37.17 (.54) | 37.27 (1.03) | .773 | .509 | .004 |
| Work Engagement (UWES) | 5.72 (.30) | 5.39 (.10) | 5.00 (.11) | 4.86 (.21) | 2.014 | .111 | .010 |
| **Vigor** | 5.59 (.33) | 5.20 (.11)^a^ | 4.63 (.12)^b^ | 4.42 (.23) | ***3.243*** | **.022** | .016 |
| Dedication | 5.80 (.34) | 5.55 (.11) | 5.25 (.13) | 5.19 (.24) | .849 | .467 | .004 |
| Absorption | 5.75 (.31) | 5.47 (.10) | 5.13 (.11) | 5.02 (.21) | 1.397 | .243 | .007 |
| Job Satisfaction | 5.77 (.39) | 5.19 (.13) | 4.89 (.15) | 5.03 (.28) | 1.445 | .229 | .007 |
| External Turnover Intention | 1.48 (.48) | 2.39 (.15) | 2.04 (.18) | 1.98 (.34) | 1.851 | .137 | .009 |
| Internal Turnover Intention | 1.48 (.55) | 2.60 (.18) | 2.64 (.20) | 2.73 (.39) | 1.522 | .208 | .007 |
| **Note.** Values are covariate-adjusted estimated marginal means (SE). Covariates held at sample means: gender, educational level, marital status, work setting, years of nursing experience, and years in current ward/unit. η²p: partial eta squared. Error df varies by model: WAI = 625, UWES = 611, Vigor/Dedication = 610, Absorption = 609, Job Satisfaction = 621, External Turnover Intention = 616, Internal Turnover Intention = 615. Bold outcome names and statistics indicate p < .05. ^a^ Generation X scored significantly higher than Millennials on Vigor after Bonferroni correction (p = .029). UWES: Utrecht Work Engagement Scale. | | | | | | | |

**Supplementary Table D**

*Effects of sociodemographic and occupational covariates across GLM models: F statistics and significance*

| **Covariate** | **WAI** | **UWES** **Total** | **Vigor** | **Dedication** | **Absorption** | **Job** **Satisfaction** | **External** **Turnover Int.** | **Internal** **Turnover Int.** |
| --- | --- | --- | --- | --- | --- | --- | --- | --- |
|  | *F (p)* | *F (p)* | *F (p)* | *F (p)* | *F (p)* | *F (p)* | *F (p)* | *F (p)* |
| **Gender** | 3.666 (.056) | .974 (.324) | 2.519 (.113) | 1.360 (.244) | .104 (.747) | 3.203 (.074) | 1.005 (.317) | .015 (.903) |
| **Educational Level** | .025 (.874) | .086 (.769) | .004 (.950) | .313 (.576) | .448 (.503) | 1.516 (.219) | ***12.796*** **(<.001)** | .665 (.415) |
| **Marital Status** | .566 (.452) | .134 (.715) | .013 (.908) | .002 (.968) | .733 (.392) | 1.000 (.318) | .014 (.907) | .690 (.407) |
| **Work Setting** **(Hospital vs Community)** | .187 (.666) | 2.994 (.084) | .659 (.417) | ***4.864*** **(.028)** | .900 (.343) | .223 (.637) | 2.083 (.149) | ***4.752*** **(.030)** |
| **Years of Nursing** **Experience** | ***16.906*** **(<.001)** | ***6.403*** **(.012)** | ***9.655*** **(.002)** | ***6.158*** **(.013)** | .988 (.321) | .924 (.337) | .564 (.453) | 2.180 (.140) |
| **Years in Current** **Ward/Unit** | ***5.816*** **(.016)** | ***6.980*** **(.008)** | ***8.187*** **(.004)** | ***6.711*** **(.010)** | 1.199 (.274) | .198 (.656) | .165 (.685) | ***4.283*** **(.039)** |
| **Note.** Each cell shows the F(1, df) statistic for the covariate in the model, with the associated p value in parentheses. All models included generational cohort as a fixed factor and all six covariates simultaneously. Bold values indicate p < .05. df values: WAI = 625; UWES = 611; Vigor = 610; Dedication = 610; Absorption = 609; Job Satisfaction = 621; External Turnover Intention = 616; Internal Turnover Intention = 615. WAI: Work Ability Index; UWES: Utrecht Work Engagement Scale. | | | | | | | | |
